# Injectable Thermoresponsive Hydrogels for Localized Drug Delivery: Mechanisms, In Vivo Evidence and Translational Challenges

**DOI:** 10.3390/jfb17090436

**Published:** 2026-09-01

**Authors:** Miriam Di Martino, Lucia Sessa, Giulia Pagliari, Daniela Silvestrino, Simona Concilio

**Affiliations:** Department of Pharmacy, University of Salerno, Via Giovanni Paolo II 132, 84084 Fisciano, SA, Italy; lucsessa@unisa.it (L.S.); gpagliari@unisa.it (G.P.); d.silvestrino@studenti.unisa.it (D.S.); sconcilio@unisa.it (S.C.)

**Keywords:** injectable hydrogel, thermoresponsive hydrogel, localized drug delivery, myocardial infarction, diabetic wound, cancer therapy

## Abstract

Injectable thermoresponsive hydrogels are useful for localized drug delivery because they can be administered as low-viscosity formulations and then form, or reinforce, therapeutic depots directly at diseased tissue sites. Their common design principle is a temperature-dependent transition from a flowable formulation before administration to an in situ matrix at physiological temperature, or a thermally regulated change in swelling, mesh size, drug-matrix affinity or degradation. This review focuses on thermoresponsive injectable systems for localized delivery, including PNIPAM-based systems, poloxamers/Pluronics, PEG/polyester block copolymers, polyurethane-based hydrogels, chitosan-based thermogels, hyaluronan- and glycosaminoglycan-based systems, and selected multicomponent or nanocomposite networks. The in vivo application areas considered are local cancer therapy, wound healing and antibacterial treatment, osteoarthritis and intra-articular delivery, and myocardial infarction/cardiac repair. Across these indications, preclinical studies suggest that thermoresponsive hydrogels may prolong local residence time, reduce systemic exposure, enhance delivery of poorly soluble or unstable payloads, and modulate disease-specific microenvironments. Remaining challenges include gelation control, mechanical stability, degradation products, immune response, sterilization, manufacturing reproducibility, disease heterogeneity and robust translational validation.

## 1. Introduction

Localized drug delivery is particularly relevant when therapeutic efficacy depends on maintaining an effective drug concentration at the target site while limiting systemic exposure. Conventional systemic administration often fails to meet this requirement because drugs may be rapidly cleared, accumulate poorly in the target tissue, or cause dose-limiting toxicity. In addition, systemic dosing rarely provides precise temporal control over drug availability at the diseased site. These limitations are particularly relevant for conditions requiring sustained local treatment, including residual tumor control after surgery, intratumoral or peritumoral therapy, infected or diabetic wounds, intra-articular inflammatory disease and post-ischemic tissue repair. More broadly, image-guided minimally invasive procedures such as embolization further illustrate the clinical relevance of locally administered materials capable of achieving site-specific therapeutic or occlusive effects [1].

Injectable hydrogels are well suited to this purpose because they can be delivered as liquids, shear-thinning networks or precursor formulations and can subsequently form or maintain a three-dimensional matrix at the site of administration. Their high water content, soft-tissue-like properties, and tunable porosity allow the incorporation of chemically diverse cargos, from small molecules and proteins to peptides, nucleic acids, nanoparticles, extracellular vesicles and cells. For peptide-based payloads, interactions with biological membranes may also influence activity and should be considered during formulation design [2,3]. Once localized in tissue, these matrices can prolong drug residence, reduce burst systemic exposure, protect labile therapeutics and provide a permissive environment for sustained or controlled release [4,5].

**Figure 1:**
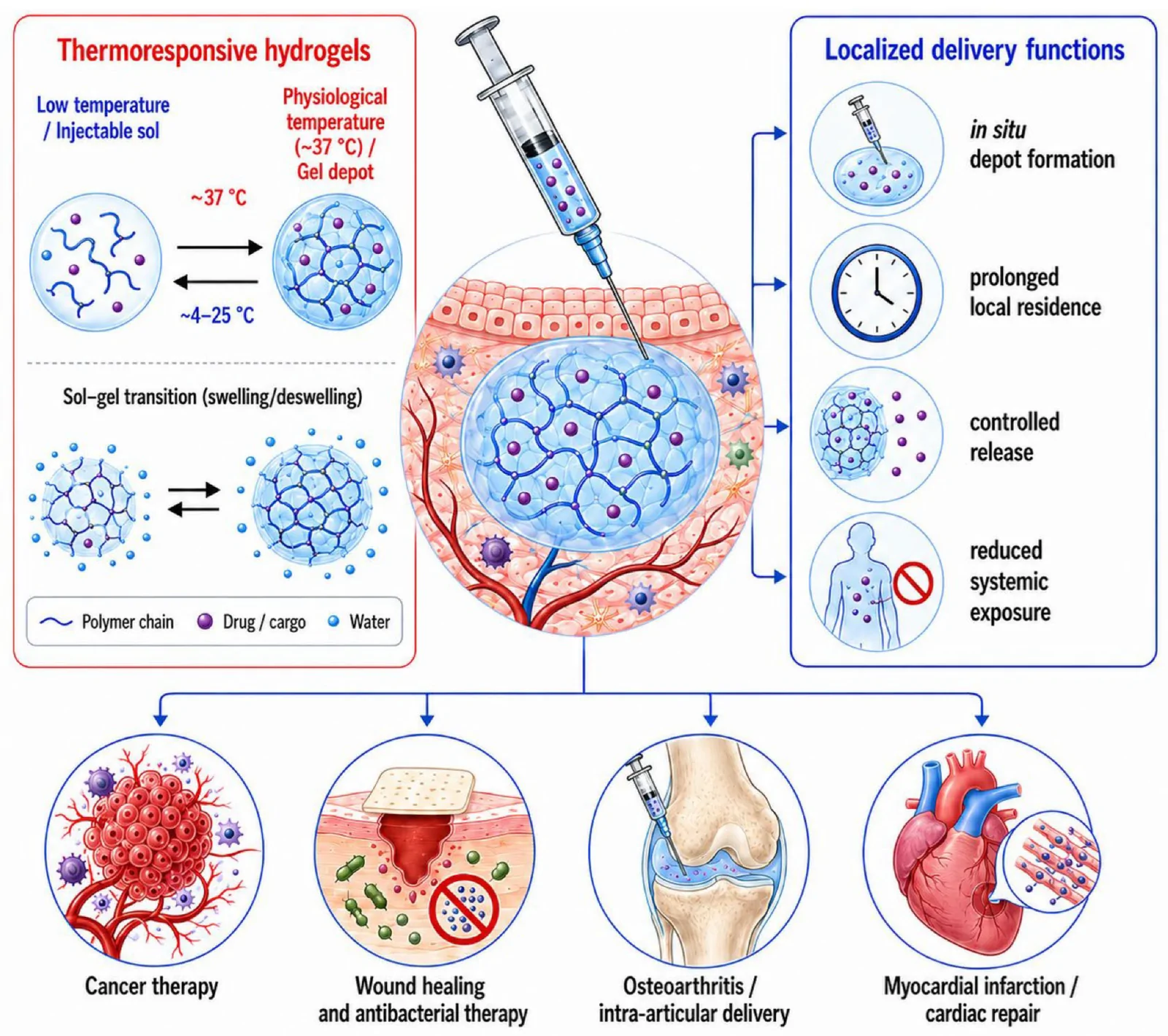
Conceptual overview of injectable thermoresponsive hydrogels for localized drug delivery. Temperature-triggered sol-gel transition enables in situ depot formation, prolonged local residence, controlled release and reduced systemic exposure across representative applications, including cancer therapy, wound/antibacterial treatment, osteoarthritis/intra-articular delivery and myocardial repair.

Thermoresponsive injectable hydrogels add a practical advantage to localized delivery: temperature can be used as a mild trigger for sol-gel transition, depot formation, swelling or deswelling, thermally regulated release, or network reinforcement. In many systems, the material remains flowable during handling and injection, and then gels upon exposure to physiological temperature [6]. Thermoresponsive delivery may rely on Lower Critical Solution Temperature (LCST) behavior, Upper Critical Solution Temperature (UCST) behavior, micellization, hydrophobic association, supramolecular assembly, or hybrid physical/chemical crosslinking [7]. In this review, the emphasis is on how temperature-responsive behavior supports injectable localized delivery rather than on polymer phase-transition theory, as depicted in Figure 1.

This review focuses on thermoresponsive injectable hydrogels and related polymer networks for localized drug delivery. Cancer therapy and wound/antibacterial treatment remain central application areas because both require site-specific therapeutic exposure and can benefit from in situ depot formation, prolonged residence and controlled release. Osteoarthritis and myocardial infarction/cardiac repair are also included because intra-articular and intramyocardial/epicardial administration are strong examples of localized delivery in clinically relevant tissues. The aim is therefore to connect material design with release behavior, in vivo evidence, biological endpoints and the translational issues that still limit the clinical use of these systems.

## 2. Literature Search and Evidence Selection

Literature was identified through a semantic, graph-based approach using VIZIT (https://www.biovista.com/vizit/, accessed on 24 June 2026). Rather than defining the corpus through a fixed keyword query, the analysis mapped relationships among the relevant biomedical concepts and their supporting publications. This approach was chosen to reduce dependence on established terminology and to capture non-obvious or less frequently reported associations. The analysis covered publications from 2015 to 2026; earlier studies, dating back to 2005, were retained when they provided foundational information on hydrogel formation or thermoresponsive polymer mechanisms. The analysis was completed in July 2026.

**Figure 2:**
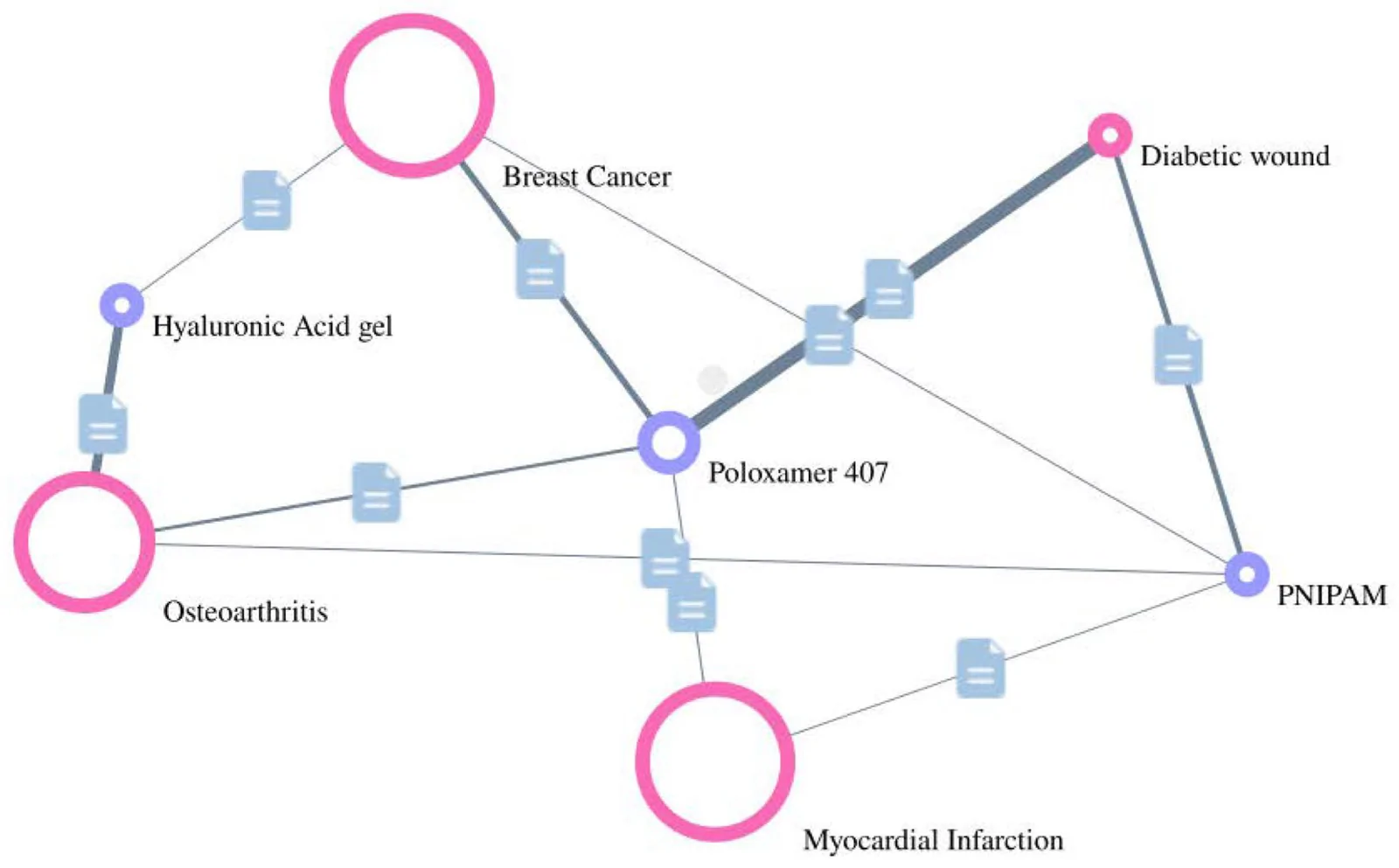
Semantic graph used for literature identification and evidence selection. The graph was generated using VIZIT and maps relationships among biomedical concepts relevant to injectable thermoresponsive hydrogels, including representative polymer platforms, localized delivery applications and disease-specific contexts. Concept nodes include materials such as Poloxamer 407, PNIPAM and hyaluronic acid gel, while application nodes include breast cancer, diabetic wound, osteoarthritis and myocardial infarction.

The semantic graph used for literature identification is shown in Figure 2 and can be viewed interactively at the following link: https://www.biovista.com/vizit/#!bv_gid=5e8dd379635414369d2efeb833b5b4da, accessed on 24 June 2026.

Publications linked to the selected graph relationships and relevant to the review scope were included; duplicates, records without traceable supporting evidence, and publications outside the defined scope were excluded.

The resulting literature corpus was analyzed using DeepSAGE, implemented within the ADAPT platform (https://adapt.softmining.it/, accessed on 24 June 2026). DeepSAGE divides publication abstracts into individual evidence statements, encodes their semantic content as numerical embeddings, and groups them using density-based clustering. Evidence synthesis is performed only after clustering: highly represented clusters contribute to the Majority Report, whereas low-weight but semantically distinct clusters contribute to the Minority Report. Unclustered statements are retained as singleton evidence units rather than discarded as noise. Consequently, infrequent, emerging, or contradictory findings are preserved rather than absorbed into the dominant narrative. Majority and minority indicate relative representation in the retrieved corpus, not scientific validity. All statements and associated citations included in the manuscript were verified by the authors.

## 3. Thermoresponsive Injectable Hydrogels for Localized Delivery

### 3.1. Mechanistic Basis: Critical Solution Temperature, Micellization and Physical Association

In this review, the term “thermoresponsive” is used to describe hydrogels whose physicochemical properties change in response to temperature; the term “thermosensitive”, when retained from the cited literature, is considered equivalent in this context. Thermoresponsive injectable hydrogels are typically designed to remain flowable before administration and to form or strengthen a depot at physiological temperature. This behavior may arise from LCST- or UCST-type phase transitions, thermally induced micellization, hydrophobic association, supramolecular interactions or reversible physical crosslinking. LCST-type systems are hydrated below the transition temperature and become less hydrated or phase-separated upon heating, whereas UCST-type systems show the opposite solubility trend. For injectable delivery, the practical requirement is that the transition or gelation window matches the intended route of administration and the target tissue environment [8,9]. Accordingly, gelation at 37 °C is a formulation-dependent design objective rather than an intrinsic feature of all thermoresponsive polymers because systems with transition temperatures below or above physiological temperature may gel prematurely or remain insufficiently gelled in vivo, respectively.

**Figure 3:**
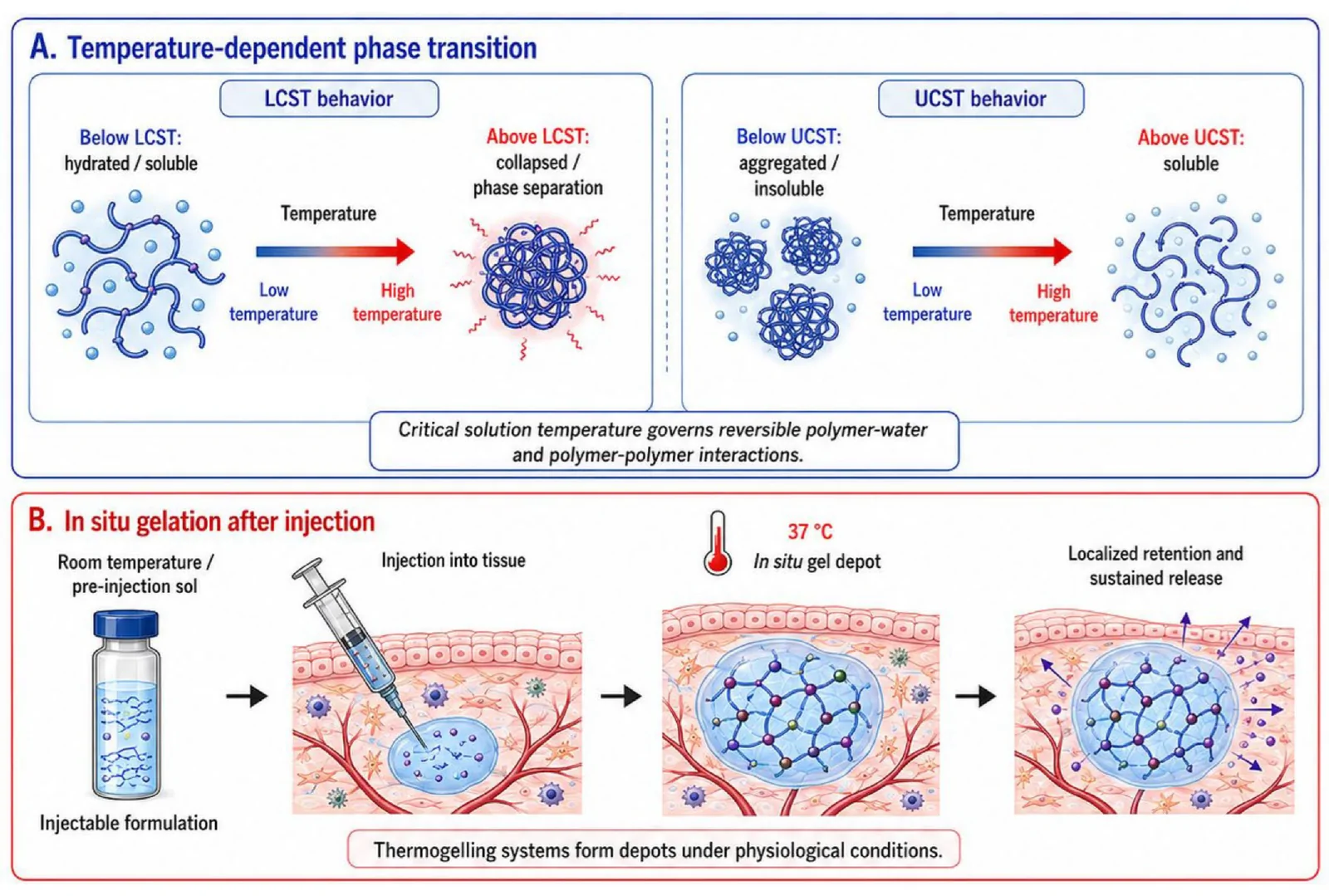
Mechanistic basis of thermoresponsive injectable hydrogels. (**A**) Temperature-dependent phase transitions showing LCST and UCST behavior and the associated changes in polymer solubility and polymer–water interactions. (**B**) In situ gelation after injection, illustrating the transition from an injectable formulation at room temperature to a gel depot under physiological conditions, enabling localized retention and sustained release.

The transition temperature and gelation profile depend on polymer composition, hydrophilic–hydrophobic balance, molecular weight, concentration, architecture, crosslinking density and the presence of salts, additives, drugs or biological fluids. Recent work on tailored thermoresponsive polyurethane hydrogels, together with studies on Pluronic/poloxamer and chitosan-based systems, illustrates how small changes in polymer architecture or formulation composition can markedly affect injectability, gelation time, mechanical reinforcement, swelling and release behavior [10,11,12]. These variables determine gelation time, depot stability, mesh size, drug release and injectability through clinically relevant needles. Figure 3 summarizes the relationship between temperature-dependent phase behavior, in situ depot formation and controlled release.

### 3.2. Thermoresponsive Polymer Platforms for Injectable Depots

Thermoresponsive injectable depots can be obtained from different polymer families, each offering a distinct balance of gelation behavior, mechanical stability, biodegradation and formulation simplicity. PNIPAM remains the prototypical LCST polymer because its phase transition in water occurs near physiological temperatures. Its hydration/dehydration transition has been widely used to tune swelling and release, including temperature- and pH-responsive PNIPAM@PAA systems for doxorubicin delivery in breast cancer models [13] and lactoferrin-targeted PNIPAM-co-acrylic acid copolymers for breast-cancer-targeted therapy [14]. However, PNIPAM has important translational limitations, particularly poor biodegradability and potential in vivo accumulation; degradable crosslinkers, copolymerization, and hybridization with natural polymers are therefore frequently explored.

Poloxamers/Pluronics are widely used amphiphilic triblock copolymers that undergo temperature- and concentration-dependent micellization followed by physical gelation. Their low viscosity at low temperatures and gelation near physiological temperatures make them attractive for minimally invasive administration. Pluronic F127 and related systems have been investigated for myocardial infarction, diabetic wounds and cancer implants [11,15,16]. Their main limitations are weak mechanical strength, rapid erosion, and limited long-term depot stability, which often motivate their combination with natural polymers such as chitosan or gelatin, inorganic or polymeric nanoparticles such as ZnO, Prussian blue nanozymes, metal–organic framework (MOF)-based particles or chitosan nanoparticles, and bioadhesive components such as hyaluronic acid, catechol-functionalized polymers or mucoadhesive polysaccharides. Secondary crosslinking strategies are also used to improve depot stability.

PEG/polyester block copolymers, including PLGA-PEG-PLGA, PEG-PLGA and PEG-PCL architectures, combine thermally induced self-assembly with tunable biodegradability. Their gelation temperature, degradation rate, mechanical properties and release behavior can be adjusted by varying block length, molecular weight and hydrophilic–hydrophobic balance [17,18].

Thermoresponsive polyurethane-based hydrogels are also relevant because segmented amphiphilic architectures can combine hydrophilic soft segments, hydrophobic or biodegradable domains, and urethane/urea hard segments capable of hydrogen bonding and reversible physical association. These systems are attractive when mechanical tunability and the ability to incorporate bioactive chain extenders or degradable soft segments are important [10,19,20].

Natural and semi-natural systems are often selected because they can provide matrix-like properties, mucoadhesion, enzymatic degradability and favorable interactions with biological tissues, while biocompatibility remains a fundamental requirement for any hydrogel intended for biomedical use. Chitosan/beta-glycerophosphate thermogels can gel near physiological temperature through reduced electrostatic repulsion, hydrogen bonding and hydrophobic association; chitosan–poloxamer and chitosan dual-network hydrogels have been investigated in cancer and diabetic wound settings [12,15,21,22]. Hyaluronan- and glycosaminoglycan-based thermoresponsive hydrogels are particularly relevant to osteoarthritis because they provide extracellular matrix-like properties, lubrication, and compatibility with cytotherapeutic or vesicular payloads [23,24,25]. Elastin-like polypeptides; poly (N-vinyl caprolactam), poly (oligoethylene glycol) methacrylates; and cellulose derivatives provide additional options with tunable transition behavior and distinct physicochemical and biomedical characteristics [26,27,28,29,30]. The main thermoresponsive polymer platforms, their characteristics and representative applications are summarized in Table 1.

### 3.3. Depot Formation, Drug Release and Characterization

Beyond polymer selection, the therapeutic performance of injectable thermoresponsive hydrogels depends on how the thermal transition translates into depot formation, local retention, and controlled release. A suitable formulation should remain injectable during handling and administration, undergo gelation or strengthening under physiologically relevant conditions, and maintain sufficient residence time at the target site. Therefore, the transition temperature alone is insufficient to predict in vivo performance. Gelation time, mechanical stability, erosion, degradation, tissue-fluid exchange, and interaction with the loaded cargo must all be considered [31].

**Figure 4:**
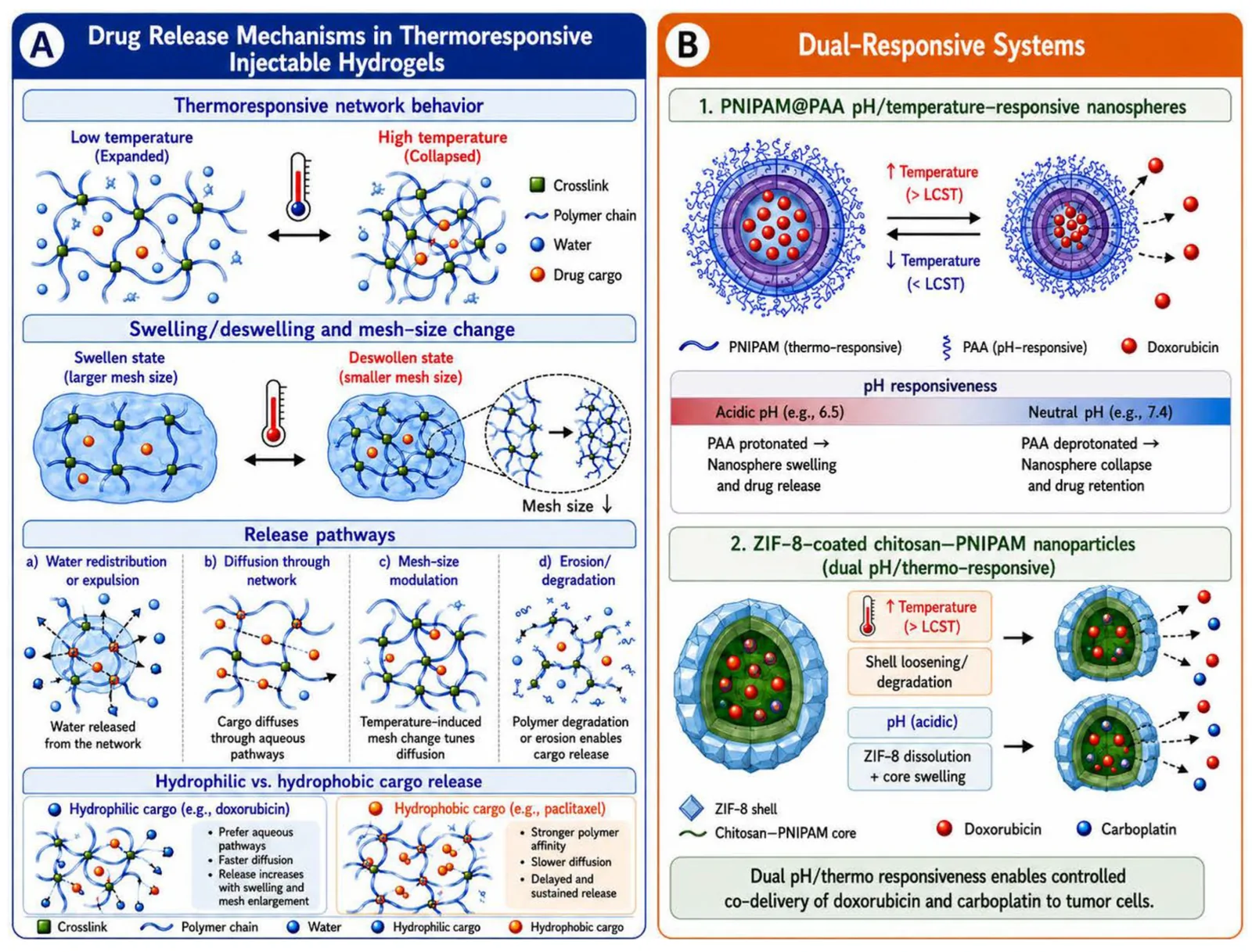
Drug release and dual-responsive behavior in thermoresponsive injectable hydrogels. (**A**) Drug release mechanisms in thermoresponsive injectable hydrogels, including temperature-induced changes in swelling, mesh size, water redistribution, drug–polymer affinity and erosion/degradation. (**B**) Dual-responsive systems integrating thermoresponsive behavior with pH-responsive release, illustrating temperature- and pH-dependent changes in polymer/nanoparticle behavior and controlled drug release.

Multiple interconnected mechanisms govern drug release from thermoresponsive hydrogels, as illustrated in Figure 4. For hydrophilic drugs, heating above the transition temperature may reduce mesh size, promote water redistribution or expulsion and alter diffusion through the network. For hydrophobic drugs, increased matrix hydrophobicity can enhance drug–polymer affinity and delay release until matrix relaxation, erosion or degradation occurs. As a result, release behavior depends on cargo solubility, polymer–drug interactions, crosslinking density, swelling/deswelling behavior, degradation kinetics and the local biological environment [32]. Multicomponent PEGDA-crosslinked networks designed for dexamethasone sodium phosphate delivery illustrate how crosslink density, hydrophilicity and swelling can be tuned to regulate corticosteroid release [33]. Corticosteroid delivery can therefore be considered a useful example of localized thermoresponsive therapy when prolonged anti-inflammatory exposure is required, while systemic glucocorticoid-related adverse effects should be minimized.

Characterization should connect material properties to depot function and release performance. FTIR can verify functional groups and the incorporation of polymers or crosslinkers; thermal analysis can assess transition behavior and stability; swelling studies provide information on water uptake and network relaxation; and rheology is essential for defining the sol-gel transition, storage modulus, shear-thinning behavior, injectability, and mechanical matching with target tissues. Release studies should ideally be performed under biologically relevant conditions and interpreted alongside degradation, swelling, and rheological data, as the same temperature-responsive transition can yield different release profiles depending on the payload and matrix composition.

Thermoresponsive systems are often combined with additional non-thermal triggers, particularly pH or redox responsiveness, when the target microenvironment provides a useful secondary cue. Examples include pH/temperature-responsive PNIPAM@PAA nanospheres for doxorubicin delivery [13] and ZIF-8-coated chitosan–PNIPAM nanoparticles for dual pH/thermoresponsive co-delivery of carboplatin and doxorubicin [34]. Such systems should be evaluated to determine whether the added responsiveness improves localized retention, release control, therapeutic efficacy, or safety compared with simpler thermoresponsive depots.

## 4. In Vivo Localized Drug Delivery Applications

Across disease models, injectable thermoresponsive hydrogels share a common therapeutic rationale: they are designed to concentrate treatment at a defined anatomical site while reducing systemic exposure. This function is particularly valuable for payloads with short half-life, poor solubility, limited tissue penetration, instability in biological fluids, or dose-limiting systemic toxicity. By forming an in situ depot after administration, thermoresponsive hydrogels can prolong local residence and provide sustained or microenvironment-adapted release of chemotherapeutics, anti-inflammatory drugs, growth factors, extracellular vesicles, antimicrobial agents, antioxidants, or cells [4,8,9,31].

The biological objective of localized delivery depends strongly on the target disease. In cancer therapy, thermoresponsive depots are mainly designed to increase local drug exposure, induce tumor cell death, reduce recurrence, and limit systemic toxicity [35]. In wound healing, they are used to control infection, regulate inflammation, promote angiogenesis, and support extracellular matrix deposition [36]. In osteoarthritis, intra-articular thermogels aim to prolong joint residence, improve lubrication, reduce inflammatory cartilage catabolism, and support chondroprotection [37]. In myocardial infarction and cardiac repair, injectable hydrogels can retain proteins, antioxidants, extracellular vesicles, or cells within the injured myocardium, promoting angiogenesis and limiting adverse remodeling [38]. These applications therefore provide complementary examples of how the same material concept, temperature-triggered depot formation, can be adapted to distinct pathological microenvironments.

### 4.1. Local Cancer Therapy

**Figure 5:**
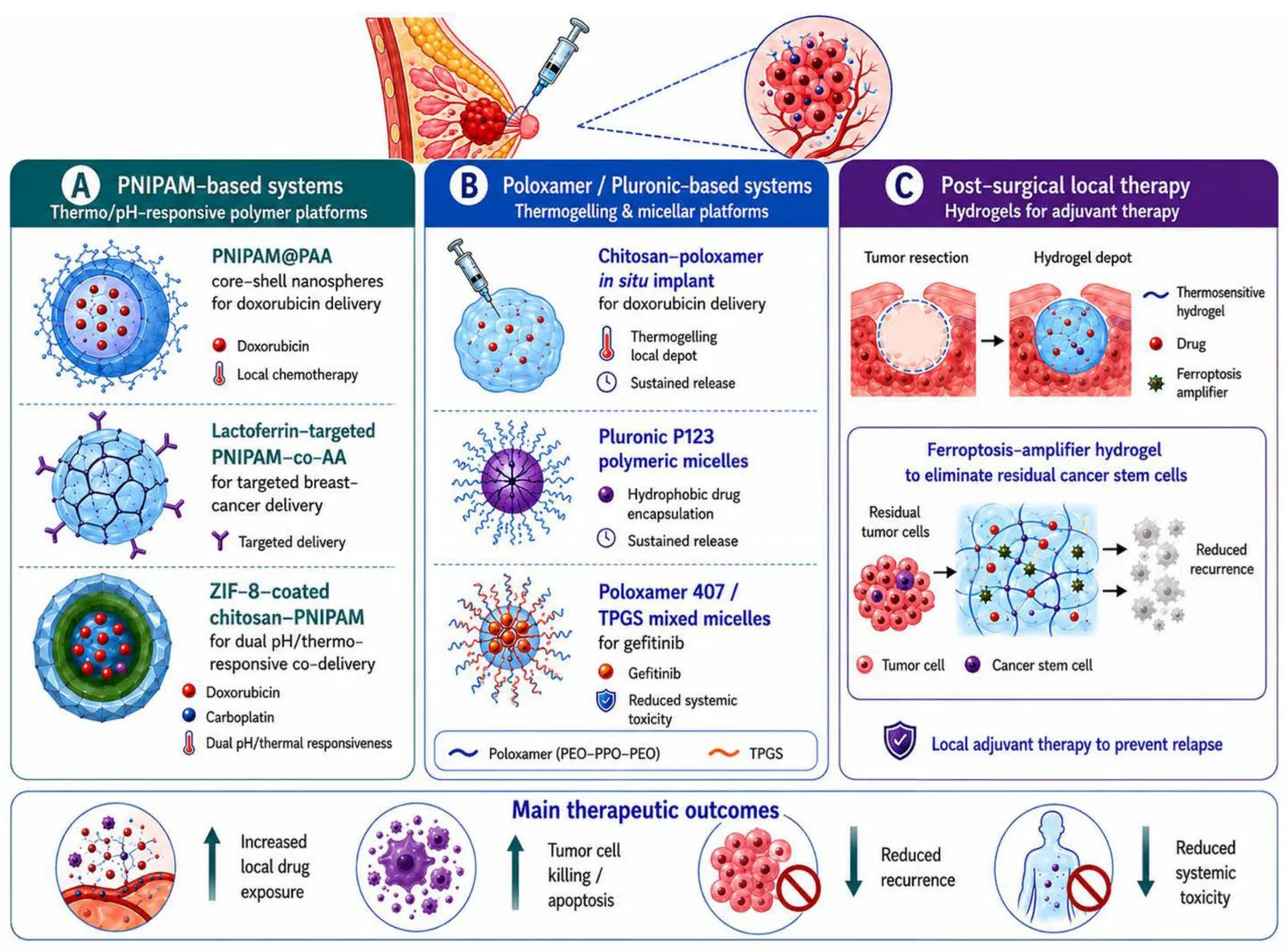
Localized cancer therapy using thermoresponsive injectable hydrogels. Representative (**A**) PNIPAM-based, (**B**) poloxamer/Pluronic-based, and (**C**) post-surgical hydrogel platforms may improve local drug exposure, support sustained or stimuli-responsive release, promote tumor cell killing and reduce recurrence or systemic toxicity.

Cancer applications use thermoresponsive systems as local depots, injectable implants, tumor-responsive nanocarriers, or microenvironment-modulating biomaterials. The therapeutic objective differs from that for regenerative indications: tumor apoptosis, ferroptosis, immune activation, and reduced proliferation or invasion are often desired, whereas systemic toxicity should be minimized. The principal systems are summarized in Figure 5.

PNIPAM-based systems are widely investigated for cancer therapy because their LCST-driven behavior can be combined with pH sensitivity, targeting ligands, or nanocarrier architectures to enable tumor-responsive drug delivery [39,40,41]. Representative examples include lactoferrin-targeted PNIPAM-co-acrylic acid copolymers developed for breast cancer therapy [14], PNIPAM@PAA nanospheres that control doxorubicin release in breast cancer treatment models [13], and ZIF-8-coated chitosan-PNIPAM nanoparticles enabling dual pH/thermoresponsive co-delivery of carboplatin and doxorubicin [34].

Poloxamer and chitosan–poloxamer formulations are also important because they can form in situ implants and increase the residence time of hydrophobic or cytotoxic drugs. Sahoo et al. [15] developed a doxorubicin-loaded chitosan–poloxamer in situ implant for breast cancer therapy. The formulation combined thermoresponsive in situ gelation with sustained doxorubicin release, cytotoxic activity and cellular uptake. It was further evaluated in vivo for pharmacokinetic behavior and anti-inflammatory activity, supporting its relevance as a localized injectable anticancer depot.

Dual ligand-targeted Pluronic P123 polymeric micelles enhanced therapy for breast cancer with bone metastases [42], while gefitinib-loaded poloxamer 407/TPGS mixed micelles provide another example of poloxamer-based cancer formulation design [43]. Post-surgical local therapy represents an additional relevant direction for thermoresponsive or in situ-forming hydrogel systems. After tumor resection, residual malignant cells within the surgical cavity can contribute to local recurrence. At the same time, systemic adjuvant therapy may be limited by toxicity and poor accumulation at the resection site. Local hydrogel depots can therefore be designed to provide sustained therapeutic exposure directly within the post-operative cavity. Zhu et al. [35] reported a ferroptosis-amplifier hydrogel designed to eliminate refractory cancer stem cells after lumpectomy. In vivo, this local post-surgical strategy reduced tumor relapse and lung metastasis, supporting the use of hydrogel depots as adjuvant platforms for eliminating residual malignant cells after tumor resection.

For local cancer therapy, the tumor microenvironment should be treated not only as a trigger for release but also as a regulator of hydrogel performance. Matrix stiffness, collagen alignment, interstitial pressure, hypoxia, acidic pH and enzymatic activity can alter depot retention and drug distribution. As reported by Passos et al. [44] and Hakariya et al. [45], collagen-binding nanoparticles for paclitaxel delivery and studies on supramolecular biomaterials that tune cancer-cell migration and cisplatin chemosensitivity further emphasize the importance of matrix affinity and material dynamics in local cancer treatment. Directional collagen cues generated by tumor-cell contraction also illustrate why matrix alignment and cell-matrix mechanics should be considered when designing local hydrogel depots [46]. Therefore, local cancer studies should report not only release kinetics and tumor volume but also intratumoral distribution, recurrence, systemic toxicity, survival, apoptosis/proliferation markers, and the fate of the injected material.

### 4.2. Wound Healing and Antibacterial Therapy

**Figure 6:**
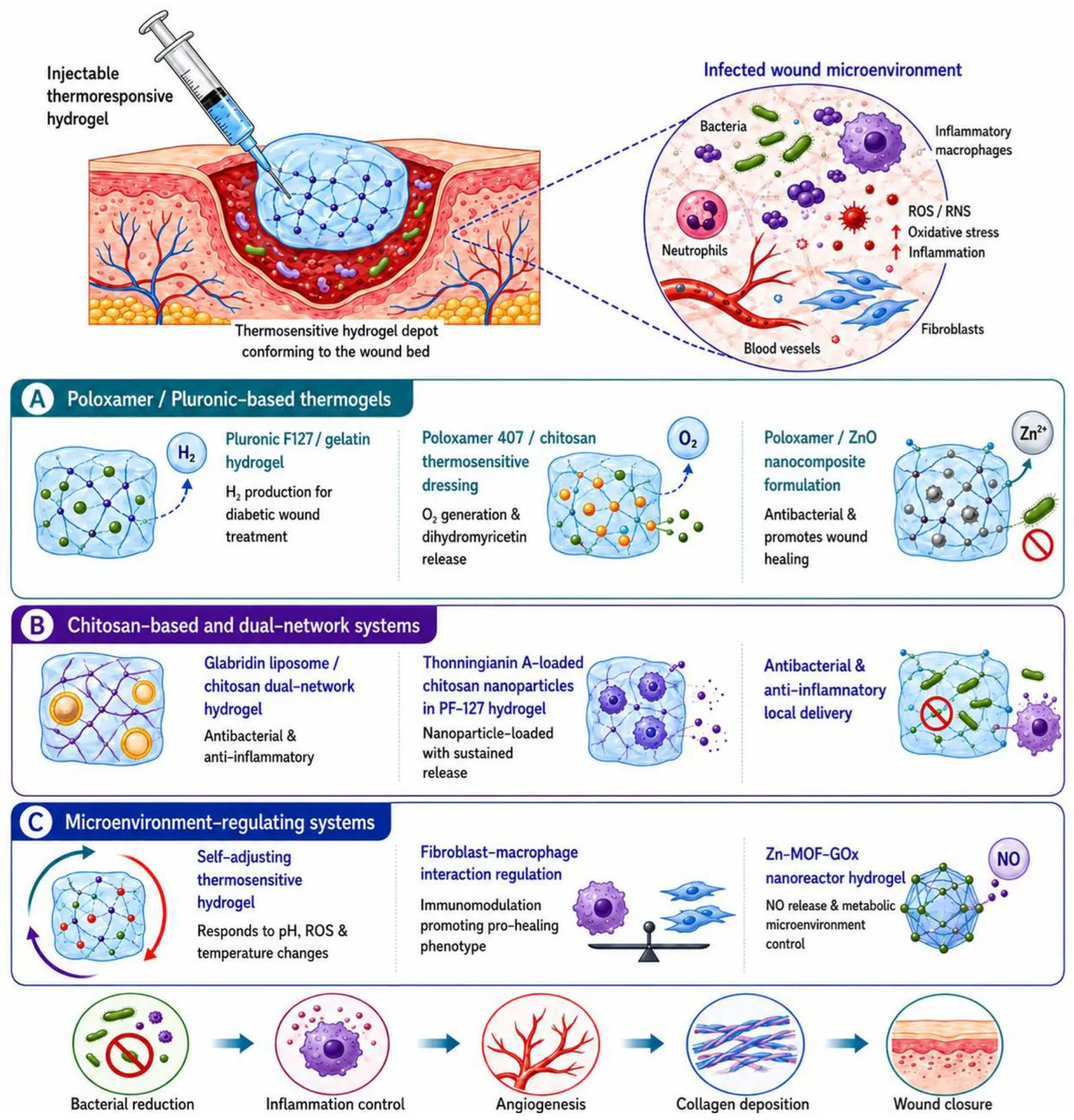
(**A**) Representative poloxamer/Pluronic-based thermogels, (**B**) chitosan-based dual-network systems and (**C**) microenvironment-regulating hydrogels can provide localized antibacterial, antioxidant, immunomodulatory and pro-regenerative effects, supporting bacterial reduction, inflammation control, angiogenesis, collagen deposition and wound closure.

Wound healing and antibacterial therapy represent natural application areas for injectable thermoresponsive hydrogels because wounds are accessible for local administration, repeated treatment and direct monitoring. Diabetic and infected wounds are characterized by chronic inflammation, oxidative stress, impaired angiogenesis, bacterial burden, dysregulated fibroblast–macrophage communication and defective matrix deposition. Thermoresponsive depots can localize antimicrobials, antioxidants, oxygen-generating components, growth-promoting molecules and immunomodulatory vesicles at the wound site (see Figure 6).

Poloxamer/Pluronic- and chitosan-based thermogels are widely used in this application area because they offer mild gelation, local retention, and compatibility with antimicrobial or antioxidant payloads. Zhang et al. [16] proposed pH-responsive injectable Pluronic F127/gelatin hydrogels producing hydrogen for diabetic wound treatment. In contrast, thermosensitive injectable glabridin liposome/chitosan dual-network hydrogels provide an example of antibacterial and anti-inflammatory delivery [12]. Liu et al. [36] developed a Poloxamer 407/chitosan thermosensitive hydrogel dressing that combines oxygen production with dihydromyricetin release to address hypoxia, oxidative stress and inflammation in diabetic wounds. In vivo, this multifunctional dressing accelerated diabetic wound repair, supporting the therapeutic value of combining local oxygen supplementation with antioxidant and anti-inflammatory modulation. Antimicrobial examples also include Thonningianin A-loaded chitosan nanoparticles encapsulated in a PF-127 hydrogel [47] and Centella asiatica extract-loaded poloxamer/ZnO nanocomposite wound closure materials [48].

Polyurethane-based thermoresponsive hydrogels have also recently been explored for diabetic wound healing. Zhai et al. [49] reported a selenium-containing polyurethane thermosensitive hydrogel for diabetic wound treatment. In vivo, this system improved wound repair by regulating oxidative stress, inflammatory responses and the local regenerative microenvironment, highlighting the potential of polyurethane-based thermogels as multifunctional wound dressings.

More complex wound systems further extend this rationale toward active microenvironment regulation rather than simple drug retention. Wound microenvironment self-adjusting hydrogels with thermosensitivity have been developed to adapt to pathological wound conditions [50].

Hydrogel-based cascade regulation of fibroblast–macrophage interactions and Zn-MOF-GOx nanoreactors, designed to promote diabetic infected wound healing through nitric oxide release and metabolic microenvironment control, further show how injectable hydrogels can coordinate antibacterial, immunomodulatory and pro-regenerative functions [51,52].

For this application area, burst release may be beneficial when rapid antimicrobial action is needed, whereas sustained or staged release is preferable for growth factors, antioxidants or immunomodulators. Key in vivo readouts include bacterial load, biofilm reduction, wound closure rate, re-epithelialization, granulation tissue thickness, collagen deposition, angiogenesis, macrophage phenotype and inflammatory cytokine levels.

### 4.3. Osteoarthritis and Intra-Articular Localized Delivery

**Figure 7:**
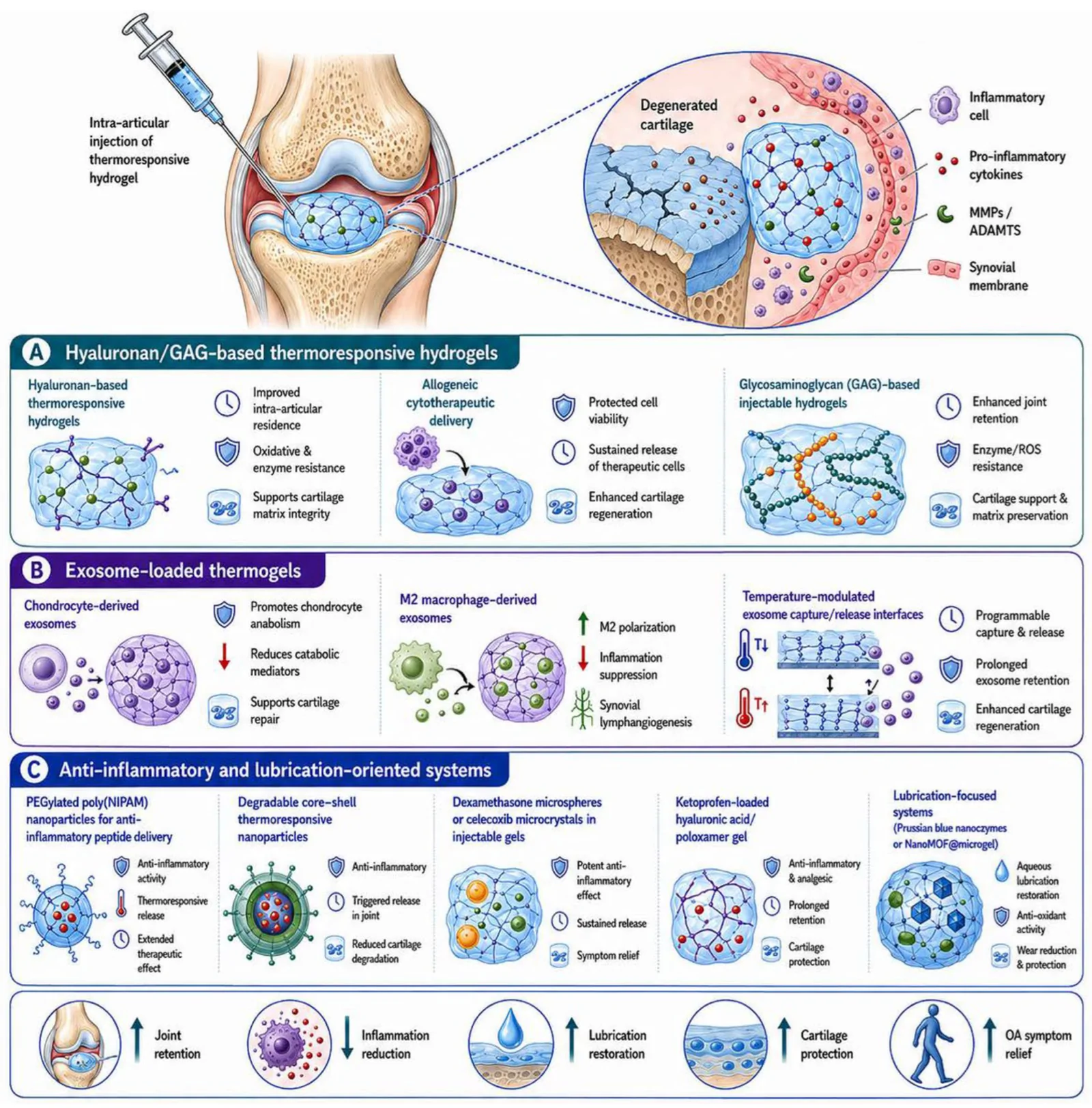
(**A**) Representative hyaluronan/GAG-based thermoresponsive hydrogels for improved intra-articular retention, cytotherapeutic delivery and cartilage-supportive functions. (**B**) Exosome-loaded thermogels for immunoregulation, chondroprotection and enhanced joint repair. (**C**) Anti-inflammatory and lubrication-oriented systems designed to reduce inflammation, protect cartilage and restore joint homeostasis.

Osteoarthritis is a particularly relevant localized delivery model because the joint cavity is accessible, local residence time is clinically important, and disease progression involves cartilage catabolism, synovial inflammation, oxidative stress, impaired lubrication and macrophage-mediated immune imbalance. The principal thermoresponsive delivery systems for osteoarthritis are summarized in Figure 7. Hyaluronan-based thermoresponsive hydrogels have been optimized for osteoarthritis management and combined with allogeneic cytotherapeutics to improve resistance to oxidative and enzymatic degradation [23,24]. Glycosaminoglycan-based injectable hydrogels have also been developed with multifunctional properties for osteoarthritis alleviation [25].

Exosome delivery represents a particularly relevant approach for intra-articular thermogels because extracellular vesicles can modulate inflammation, macrophage phenotype and cartilage repair, while requiring protection and retention within the joint cavity. Sang et al. [53] showed that thermosensitive hydrogels loaded with primary chondrocyte-derived exosomes promoted cartilage repair in vivo by regulating macrophage polarization toward a more reparative phenotype. Similarly, Song et al. [37] reported that injectable thermosensitive hydrogels delivering M2 macrophage-derived exosomes attenuated osteoarthritis progression in vivo by promoting synovial lymphangiogenesis and improving the synovial lymphatic microenvironment.

Temperature-modulated exosome capture and release interfaces provide a related technological basis for designing exosome-compatible thermoresponsive platforms [54].

Anti-inflammatory delivery and lubrication form a second major intra-articular axis. Hollow PEGylated poly (NIPAM) nanoparticles can deliver anti-inflammatory peptides in ex vivo osteoarthritis models [55], and degradable core-shell thermoresponsive nanoparticles have been developed for intra-articular anti-inflammatory peptide delivery [56].

Dexamethasone microspheres and celecoxib microcrystals loaded into injectable gels have been explored to enhance therapy for knee osteoarthritis [57]. In contrast, ketoprofen-loaded transethosomes in hyaluronic acid/poloxamer gel provide a further example of local anti-inflammatory formulation design [58]. Lubrication-focused thermoresponsive systems, including hybrid osteoarthritis drug-delivery nanocarriers, Pluronic-coated Prussian blue nanozymes, core-shell NanoMOFs@microgel systems and dual-functional MOF-based hybrid microgels, further show how intra-articular formulations can combine aqueous lubrication, thermally responsive release and anti-inflammatory activity [59,60,61,62].

Overall, these studies indicate that intra-articular thermoresponsive systems should be evaluated not only as drug depots but also as materials that influence joint residence, lubrication, inflammation, and cartilage preservation.

### 4.4. Myocardial Infarction and Cardiac Repair

Myocardial infarction represents a demanding localized delivery context because materials must be retained within mechanically active tissue while supporting angiogenesis, cardiomyocyte survival, inflammation control and fibrosis reduction. Intramyocardial or epicardial thermoresponsive hydrogels have therefore been explored as local depots for proteins, growth factors, antioxidants, anti-inflammatory drugs, extracellular vesicles and cells.

Lee et al. reported an injectable sulfonated reversible thermal gel for therapeutic angiogenesis after myocardial infarction [63], while Rocker et al. developed a sulfonated thermoresponsive injectable gel for sequential release of therapeutic proteins, including VEGF, IL-10 and PDGF, to protect cardiac function after infarction [38].

Growth-factor-loaded thermosensitive hydrogels provide some of the most concrete in vivo evidence in myocardial repair. In a VEGF165 study, intramyocardial delivery using a biodegradable hydrogel promoted angiogenesis and improved cardiac function after myocardial infarction [64]. Zhu et al. [65] further showed that bFGF delivery through a Dex-PCL-HEMA/PNIPAAm thermosensitive hydrogel enhanced angiogenesis, reduced collagen deposition, infarct area and apoptosis, and provided additional functional benefit compared with either bFGF or hydrogel alone. Alginate-chondroitin sulfate in situ-gelling scaffolds loaded with platelet growth factors improved cardiomyocyte survival after ischemia [66], while HGF-associated extracellular vesicle microcarriers extended this strategy toward combined paracrine and vesicular therapy [67].

Drug-loaded and multifunctional cardiac hydrogels include emodin-loaded Pluronic F127 hydrogels designed to improve efficacy and reduce hepatotoxicity [11], TPL@PLGA@F127 thermoresponsive hydrogels proposed for localized myocardial infarction therapy to reduce the systemic hepato- and nephrotoxicity associated with free triptolide [68], and Dex-PCL-HEMA/PNIPAAm (DPHP) thermoresponsive hydrogels reported to inhibit post-infarct heart failure in rat models [69].

A parallel strategy involves conductive or mechanically adaptive platforms aimed at improving electrical integration, structural support or regenerative signaling. These include thermosensitive flexible hydrogels capable of supporting cardiac differentiation under mechanical training [70], PNIPAAm-based biohybrid injectable hydrogels for cardiac tissue engineering [71], injectable PNIPAAm hydrogels containing niosomal angiogenic drug delivery systems [72], electroconductive chitosan/Pluronic/gold-decorated cellulose nanofiber hydrogels [73], and injectable conductive nanomicelle hydrogels containing alpha-tocopherol for myocardial infarction repair [74].

**Figure 8:**
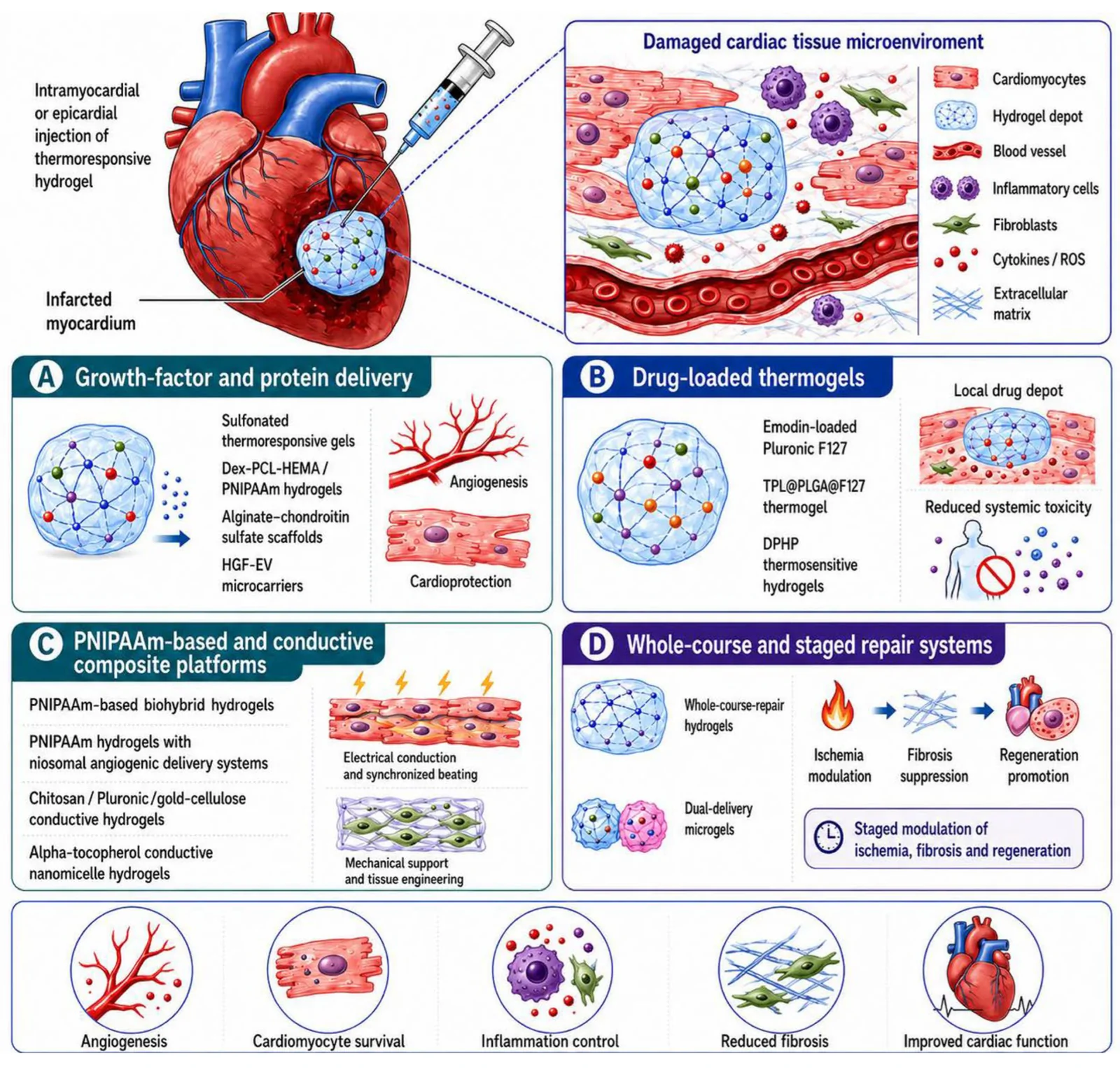
Myocardial infarction and cardiac repair using injectable thermoresponsive hydrogels. (**A**) Growth-factor and protein delivery using representative thermoresponsive platforms for angiogenesis and cardioprotection. (**B**) Drug-loaded thermogels for localized drug delivery and sustained release. (**C**) PNIPAAm-based and conductive composite platforms supporting electrical conduction, synchronized beating and tissue engineering. (**D**) Whole-course and staged repair systems designed to modulate ischemia, suppress fibrosis and promote cardiac regeneration. Together, these platforms may support angiogenesis, cardiomyocyte survival, inflammation control, reduced fibrosis and improved cardiac function.

Whole-course-repair stimulus-responsive hydrogels and dual-delivery microgel therapeutics further illustrate the shift from single-payload depots toward staged modulation of ischemia, fibrosis and regeneration [75,76]. At the same time, negative or neutral findings, such as the absence of a direct postconditioning effect of Poloxamer 188 on mitochondrial function after ischemia-reperfusion injury, indicate that studies should distinguish material-mediated retention or mechanical benefit from direct pharmacological effects [77]. Figure 8 summarizes the most representative myocardial infarction and cardiac repair injectable thermoresponsive hydrogels.

In all these areas of application, thermoresponsive injectable hydrogels share the common objective of improving local retention and controlling payload release, but the dominant design requirements differ according to the target tissue and therapeutic goal. In cancer therapy, the main priority is sustained local exposure and recurrence control after intratumoral, peritumoral or post-surgical administration. In wound healing, hydrogel performance is more closely linked to antibacterial activity, inflammation control, oxygen balance, angiogenesis and matrix deposition. In osteoarthritis, intra-articular residence, lubrication and cartilage protection are central requirements, whereas in myocardial infarction and cardiac repair, mechanical compatibility, retention in contractile tissue, angiogenesis, cardiomyocyte survival and fibrosis control are particularly important. These differences indicate that thermoresponsive depot design should be application-specific rather than based only on generic sol-gel behavior.

A comparative summary of the representative thermoresponsive systems discussed in the application sections is provided in Table 2, including the main platforms, therapeutic payloads or functions, localized delivery rationale and representative references.

## 5. Common Findings and Less Common or Debated Findings

A recurring theme in the selected literature is that injectable thermoresponsive hydrogels can enhance local therapeutic exposure by prolonging residence time and enabling controlled or sustained release of drugs, proteins, peptides, extracellular vesicles, cells and nanotherapeutics. This advantage is most convincing when the target site is accessible to local administration and when systemic therapy is limited by toxicity, short half-life, poor solubility or insufficient tissue penetration. Cancer therapy, diabetic wound healing, osteoarthritis and myocardial infarction all meet these criteria in different ways.

A second common finding is that thermoresponsive hydrogels rarely act solely as passive drug depots. In many studies, they also modulate the local microenvironment by influencing inflammation, angiogenesis, oxidative stress, extracellular matrix remodeling, macrophage polarization, fibrosis, apoptosis or tissue retention of biologics. This microenvironmental role is particularly evident in cardiac repair, osteoarthritis and wound healing, where the therapeutic objective is not only payload release but also the restoration of a more favorable repair or regeneration niche.

Less common or still-debated findings concern long-term biocompatibility, biodegradability, immune response, degradation products, reproducibility of synthesis, and translation of complex multicomponent formulations. These limitations are particularly relevant for systems containing non-degradable synthetic polymers, inorganic nanoparticles, conductive fillers, MOFs, biologics, or multiple interacting components [5]. Patient- and disease-related variability also remains important, as disease stage, vascularization, inflammatory tone, enzymatic activity, oxidative stress, and local tissue mechanics can alter hydrogel degradation, retention, and therapeutic response.

Another debated point is whether increasing formulation complexity consistently translates into superior in vivo efficacy. Dual-responsive nanoparticles, nanocomposite gels, biologic-loaded depots, conductive networks and staged-release systems may offer mechanistic advantages, but they also increase manufacturing, sterilization, and regulatory and safety burdens [4]. Comparative studies against simpler thermoresponsive depots are therefore needed to determine when added responsiveness, conductivity, nanostructuring or multi-payload delivery is truly justified.

## 6. Preclinical Evaluation

Preclinical evaluation should connect material behavior with disease-specific biological endpoints and should be conducted and reported according to rigorous in vivo experimental design principles [78].

In the studies reviewed, preclinical assessment generally includes administration route, depot-forming behavior, release profile, therapeutic efficacy and selected local biological endpoints. However, the depth of reporting varies substantially across applications. Long-term depot persistence, degradation, biodistribution, systemic fate and the contribution of individual formulation components are less consistently documented.

Several studies include controls such as free payloads, blank hydrogels, or unloaded carriers, allowing the therapeutic contribution of the depot to be partially distinguished from that of the delivered drug, cell, vesicle, or nanoparticle. However, direct comparisons with non-thermoresponsive formulations, simpler thermoresponsive depots, or individual components of multicomponent systems are not always conducted. This remains important for dual-responsive nanoparticles, conductive hydrogels, MOF-containing systems, biologics-loaded depots, and staged-release platforms, where therapeutic benefit may arise from the payload, the material, or their combined interaction with the local microenvironment.

Because localized therapy aims to increase residence time and reduce systemic exposure, biodistribution and safety are critical but unevenly documented in the reviewed literature. Local histology, inflammatory response, tissue repair markers and systemic toxicity indicators are frequently reported. In contrast, the long-term fate of polymer fragments, nanoparticles, residual reagents or released payloads is less often examined. These aspects should be evaluated more systematically, particularly for formulations intended for repeated administration or long-term depot formation.

Disease-specific efficacy endpoints are typically selected to align with the biological objective of each application. In cancer studies, common endpoints include tumor suppression, recurrence, survival, intratumoral distribution and systemic toxicity. Wound studies frequently assess infection control, re-epithelialization, angiogenesis, collagen deposition and inflammatory resolution. Osteoarthritis studies assess joint retention, lubrication, cartilage preservation, synovial inflammation and macrophage polarization, whereas myocardial infarction studies evaluate cardiac function, infarct size, angiogenesis, cardiomyocyte survival, inflammation and fibrosis. Application-specific biological endpoints and representative readouts are summarized in Table 3.

## 7. Translational Challenges and Future Perspectives

Despite extensive preclinical progress, the clinical translation of injectable thermoresponsive hydrogels for localized drug delivery remains limited by formulation, biological, manufacturing and regulatory challenges [5,8]. Accordingly, the encouraging results obtained in preclinical models should be interpreted as evidence of therapeutic potential rather than as direct evidence of clinical efficacy. Clinically relevant systems should remain injectable before administration, avoid premature gelation during handling and form a stable depot after injection to prevent leakage, dilution or systemic dispersion. Because tumors, wounds, joints and infarcted myocardium differ in fluid exchange, enzymatic activity, vascularization and mechanical loading, gelation temperature, viscosity, modulus, swelling behavior, degradation and release kinetics should be optimized according to the target tissue rather than treated as universal parameters.

Biocompatibility and degradation remain central issues. Hydrogel composition and degradation products may influence local inflammatory, hypersensitivity, or fibrotic responses, while the long-term fate of non-degradable components should be carefully assessed [8]. These concerns are particularly relevant for PNIPAM-based systems, inorganic nanoparticle-containing hydrogels, MOF-based formulations, conductive composites, multicomponent nanogels and biologic-loaded depots. Systemic exposure to polymer fragments, nanoparticles, residual reagents or released drugs should also be assessed, especially for highly vascularized or mechanically active tissues.

Manufacturing, sterilization and storage represent additional barriers. Thermoresponsive behavior is sensitive to polymer molecular weight, concentration, monomer ratio, hydrophilic–hydrophobic balance, crosslink density and temperature history. Small variations in these parameters can alter sol-gel transition, injectability, depot stability and release kinetics. Sterilization and storage may further affect polymer architecture, degradation profile, drug stability and biological payload activity, particularly for hydrogels carrying proteins, extracellular vesicles, cells or labile small molecules [79].

Future development should balance multifunctionality with translational simplicity. Dual-responsive nanoparticles, conductive networks, MOF-containing systems, oxygen-generating hydrogels, immunomodulatory depots, and staged-release platforms may offer mechanistic advantages but also increase the manufacturing, quality control, and regulatory burden [4,5]. The most promising thermoresponsive hydrogels will likely be those that combine reliable injectability, predictable gelation, controlled degradation, reproducible release, acceptable safety and clear superiority over free drug administration or simpler local depots. Standardized preclinical reporting, imaging-based depot tracking and data-driven formulation optimization could further accelerate translation toward application-specific localized therapy.

## 8. Conclusions

Injectable thermoresponsive hydrogels are attractive for localized drug delivery because they combine minimally invasive administration with in situ depot formation and sustained or thermally regulated release. Their value is greatest when the target site is accessible and when systemic therapy is limited by toxicity, poor solubility, a short half-life or insufficient tissue accumulation. Across cancer therapy, wound and antibacterial treatment, osteoarthritis and myocardial repair, the same material principles can be adapted to different biological goals, including tumor suppression, infection control, inflammation resolution, cartilage preservation, angiogenesis and tissue remodeling.

The field should now move beyond proof-of-concept thermogelation towards rigorous evaluation of material composition, depot persistence, release behavior, degradation, local immune response and disease-specific efficacy. Future progress will depend on demonstrating not only attractive polymer chemistry and payload compatibility but also reproducible manufacturing, predictable safety and a clear therapeutic advantage over free-drug administration or simpler local depots. These criteria will determine whether thermoresponsive injectable hydrogels may progress towards clinically relevant localized therapy.

## Figures and Tables

**Table 1:** Representative thermoresponsive polymer platforms for injectable localized drug delivery.

Thermoresponsive Platform	Main Mechanism	Typical Behavior	Main Advantages	Main Limitations	Representative Localized Applications
PNIPAM-based hydrogels	LCST-driven dehydration, hydrophobic association and chain collapse	Swollen below LCST; collapsed/deswollen above LCST; transition tunable by copolymerization	Sharp reversible transition; well-established model; easy modification	Poor biodegradability; possible accumulation; safety/clearance concerns	Triggered release, cancer therapy, responsive depots
Poloxamers/Pluronics	Temperature- and concentration-dependent micellization followed by physical gelation	Low-viscosity sol at low temperature; gel near physiological temperature	Simple formulation; good injectability; mild gelation; pharmaceutical familiarity	Weak mechanics; rapid erosion; limited long-term depot stability	Cancer implants, diabetic wounds, cardiac delivery, local drug delivery
PEG/polyester block copolymers	Thermally induced micellization, aggregation and physical network formation	Sol-gel transition near physiological temperature; degradation controlled by polyester blocks	Tunable degradation; synthetic flexibility; sustained release	Possible acidic degradation products; batch reproducibility; erosion control	Sustained local delivery, post-operative delivery, tissue repair
Polyurethane-based hydrogels	Temperature-dependent micellization, hydrophobic association, hydrogen bonding and reversible physical crosslinking	Injectable sol or shear-thinning network that strengthens/gels depending on soft/hard segment composition	Highly tunable chemistry, adjustable mechanics, degradable segments and bioactive chain extenders are possible	Design-dependent thermoresponsiveness; complex synthesis; residual isocyanates/catalysts and degradation products require control	Localized drug delivery, wound healing, anticancer depots, regenerative/tissue repair applications
Chitosan/beta-glycerophosphate and chitosan–poloxamer systems	Reduced electrostatic repulsion, hydrogen bonding, hydrophobic association and physical gelation	Injectable at low temperature; gelation around physiological temperature	Biocompatibility; mucoadhesion; biodegradability; antibacterial potential	Batch variability; limited mechanics; gelation sensitive to pH/ionic strength	Wound healing, antibacterial delivery, cancer implants, anti-inflammatory delivery
Hyaluronan and glycosaminoglycan hydrogels	ECM-like network formation with thermoresponsive or hybrid physical association	Injectable viscoelastic systems; tunable degradation/lubrication properties	Matrix mimicry; joint lubrication; cytotherapeutic compatibility	Rapid degradation unless stabilized; batch variability; mechanical limitations	Osteoarthritis, intra-articular delivery, cartilage repair
Elastin-like polypeptides	Sequence-defined temperature-triggered coacervation or phase transition	Soluble below transition; self-assembly/coacervation above transition	Genetically tunable; biodegradable; protein-like architecture	Production cost, scalability, and immunogenicity assessment are required	Protein delivery, regenerative depots
Poly (N-vinyl caprolactam) and POEGMA systems	LCST-type hydration/dehydration tuned by composition and side chains	Thermally regulated swelling/deswelling near selected temperatures	Tunable transition; non-ionic options; potentially lower toxicity than PNIPAM	Less established; degradation and long-term clearance require evaluation	Controlled release, injectable depots, multi-responsive systems
Cellulose or other polysaccharide derivatives	Temperature-dependent hydrophobic association and physical gelation depending on substitution pattern	Thermogelation or viscosity increase with temperature	Natural/semi-natural origin; mild formulation; biocompatibility	Broad transitions; weaker mechanics; limited degradation control	Wound healing, mucosal/local delivery, antibacterial delivery

**Table 2:** Representative examples of injectable thermoresponsive hydrogels and related polymer systems for localized therapy.

Application	Representative Systems	Main Payload/Function	Therapeutic Rationale	Representative References
Localized cancer therapy	PNIPAM@PAA nanospheres; lactoferrin-targeted PNIPAM-co-AA copolymers; ZIF-8-coated chitosan-PNIPAM nanoparticles; chitosan–poloxamer in situ implants; Pluronic P123 and poloxamer 407/TPGS micelles; post-surgical ferroptosis-amplifier hydrogels.	Doxorubicin; carboplatin/doxorubicin co-delivery; targeted chemotherapy; gefitinib; local ferroptosis-based adjuvant therapy.	Increase local tumor drug exposure; support tumor-responsive release; reduce recurrence and limit systemic toxicity.	[13,14,15,34,35,43]
Wound healing and antibacterial therapy	Pluronic F127/gelatin hydrogels; chitosan/poloxamer systems; PF-127-loaded chitosan nanoparticle hydrogels; selenium-containing polyurethane thermoresponsive hydrogels; microenvironment-regulating hydrogels.	Hydrogen/oxygen production; antimicrobial/anti-inflammatory delivery; antioxidant and microenvironment-modulating activity.	Control infection, oxidative stress, hypoxia and inflammation; promote angiogenesis, collagen deposition and wound closure.	[12,16,36,47]
Osteoarthritis and intra-articular delivery	Hyaluronan thermoresponsive hydrogels; glycosaminoglycan-based injectable hydrogels; exosome-loaded thermosensitive hydrogels; PEGylated PNIPAM nanoparticles; HA/poloxamer gels; Prussian blue/Pluronic nanozymes; NanoMOFs@microgel and MOF-based hybrid microgels.	Cytotherapeutics; exosomes; anti-inflammatory peptides/drugs; lubrication-enhancing systems; antioxidant/nanozyme activity.	Increase intra-articular retention; improve lubrication; reduce inflammation and oxidative stress; and support cartilage protection.	[23,24,25,37,53]
Myocardial infarction/cardiac repair	Sulfonated thermoresponsive gels; Dex-PCL-HEMA/PNIPAAm hydrogels; alginate-chondroitin sulfate in situ-gelling scaffolds; Pluronic F127 and TPL@PLGA@F127 thermogels; DPHP thermoresponsive hydrogels; PNIPAAm-based injectable hydrogels; chitosan/Pluronic/gold-cellulose conductive hydrogels; staged microgel systems.	VEGF/IL-10/PDGF; VEGF165; bFGF; platelet growth factors; emodin; triptolide; conductive/regenerative support; staged ischemia/fibrosis/regeneration modulation.	Retain therapeutics in injured myocardium; promote angiogenesis and cardiomyocyte survival; reduce inflammation/fibrosis; and improve cardiac function.	[38,64,65,68,73]

**Table 3:** Application-specific biological endpoints and representative readouts for preclinical evaluation of injectable thermoresponsive hydrogels.

Application	Hydrogel-Linked Endpoint	Representative Readouts	Representative References
Cancer therapy	Tumor apoptosis/ferroptosis, reduced proliferation and invasion, immune activation, reduced systemic toxicity.	Tumor volume, recurrence, survival, histology, CASP3/BAX/BCL2, Ki-67, VEGFA/HIF1A, MMP2/MMP9, immune-cell infiltration, systemic toxicity markers.	[13,14,15,34,35,40,41,42,43,44]
Wound/antibacterial therapy	Resolution of chronic inflammation, bacterial reduction, angiogenesis, collagen deposition and re-epithelialization.	Wound closure, bacterial load, biofilm disruption, CD31/VEGFA, IL-1β/TNF-α/IL-6, COL1A1/COL3A1, macrophage markers.	[12,16,36,47,48,49,50,52]
Osteoarthritis	Chondroprotection, lubrication, macrophage polarization, reduced cartilage catabolism and synovial inflammation.	Cartilage histology, OARSI score, SOX9, COL2A1, ACAN, PRG4, MMP13, ADAMTS5, IL-1β/TNF-α, CD206/ARG1.	[23,24,25,37,53,54,55,56,57,58]
Myocardial infarction/cardiac repair	Angiogenesis, cardiomyocyte survival, anti-inflammatory repair and reduced fibrosis/remodeling.	Ejection fraction, infarct size, ventricular remodeling, CD31/VEGFA, FGF2, IL-10, TNF-α, COL1A1/COL3A1, ACTA2, CASP3/BAX/BCL2.	[38,63,64,65,66,67,71,72,73,76]

## Data Availability

No new data were created or analyzed in this study. Data sharing is not applicable to this article.

## Abbreviations

The following abbreviations are used in this manuscript:

AA	Acrylic acid
ACAN	Aggrecan
ACTA2	Actin alpha 2, smooth muscle
ADAMTS5	A disintegrin and metalloproteinase with thrombospondin motifs 5
ARG1	Arginase 1
BAX	BCL2-associated X protein
BCL2	B-cell lymphoma 2
bFGF	Basic fibroblast growth factor
CASP3	Caspase 3
CD31	Cluster of differentiation 31
CD206	Cluster of differentiation 206
COL1A1	Collagen type I alpha 1 chain
COL2A1	Collagen type II alpha 1 chain
COL3A1	Collagen type III alpha 1 chain
Dex-PCL-HEMA/PNIPAAm	Dextran-polycaprolactone-2-hydroxyethyl methacrylate/poly(N-isopropylacrylamide)
ECM	Extracellular matrix
F127	Pluronic F127
FGF2	Fibroblast growth factor 2
FTIR	Fourier-transform infrared spectroscopy
GAG	Glycosaminoglycan
GOx	Glucose oxidase
HA	Hyaluronic acid
HEMA	2-Hydroxyethyl methacrylate
HGF	Hepatocyte growth factor
HIF1A	Hypoxia-inducible factor 1 alpha
IL-1β	Interleukin-1 beta
IL-6	Interleukin-6
IL-10	Interleukin-10
M2	M2 macrophage phenotype
MMP2	Matrix metalloproteinase 2
MMP9	Matrix metalloproteinase 9
MMP13	Matrix metalloproteinase 13
MOF/MOFs	Metal–organic framework(s)
NanoMOFs	Nanoscale metal–organic frameworks
NO	Nitric oxide
OARSI	Osteoarthritis Research Society International
P123	Pluronic P123
PAA	Poly(acrylic acid)
PCL	Polycaprolactone
PDGF	Platelet-derived growth factor
PEG	Polyethylene glycol
PEGDA	Polyethylene glycol diacrylate
PF-127	Pluronic F127
PLGA	Poly(lactic-co-glycolic acid)
PNIPAM/PNIPAAm	Poly(N-isopropylacrylamide)
POEGMA	Poly(oligoethylene glycol) methacrylate
PRG4	Proteoglycan 4
SOX9	SRY-box transcription factor 9
TNF-α	Tumor necrosis factor alpha
TPGS	D-α-tocopheryl polyethylene glycol succinate
TPL	Triptolide
VEGF	Vascular endothelial growth factor
VEGF165	Vascular endothelial growth factor 165
VEGFA	Vascular endothelial growth factor A
ZIF-8	Zeolitic imidazolate framework-8
Zn-MOF-GOx	Zinc metal–organic framework–glucose oxidase system
ZnO	Zinc oxide
